# Valproic acid-exposed astrocytes impair inhibitory synapse formation and function

**DOI:** 10.1038/s41598-020-79520-7

**Published:** 2021-01-08

**Authors:** Kotomi Takeda, Takuya Watanabe, Kohei Oyabu, Shuntaro Tsukamoto, Yuki Oba, Takafumi Nakano, Kaori Kubota, Shutaro Katsurabayashi, Katsunori Iwasaki

**Affiliations:** 1 Department of Neuropharmacology, Faculty of Pharmaceutical Sciences, Fukuoka University, Fukuoka, 814-0180 Japan; 2 A.I.G. Collaborative Research Institute for Aging and Brain Sciences, Fukuoka University, Fukuoka, 814-0180 Japan; 3 Department of Pharmaceutical and Health Care Management, Faculty of Pharmaceutical Sciences, Fukuoka University, Fukuoka, 814-0180 Japan

**Keywords:** Neurology, Cellular neuroscience

## Abstract

Valproic acid (VPA) is widely prescribed to treat epilepsy. Maternal VPA use is, however, clinically restricted because of the severe risk that VPA may cause neurodevelopmental disorders in offspring, such as autism spectrum disorder. Understanding the negative action of VPA may help to prevent VPA-induced neurodevelopmental disorders. Astrocytes play a vital role in neurodevelopment and synapse function; however, the impact of VPA on astrocyte involvement in neurodevelopment and synapse function has not been examined. In this study, we examined whether exposure of cultured astrocytes to VPA alters neuronal morphology and synapse function of co-cultured neurons. We show that synaptic transmission by inhibitory neurons was small because VPA-exposed astrocytes reduced the number of inhibitory synapses. However, synaptic transmission by excitatory neurons and the number of excitatory synapses were normal with VPA-exposed astrocytes. VPA-exposed astrocytes did not affect the morphology of inhibitory neurons. These data indicate that VPA-exposed astrocytes impair synaptogenesis specifically of inhibitory neurons. Our results indicate that maternal use of VPA would affect not only neurons but also astrocytes and would result in perturbed astrocyte-mediated neurodevelopment.

## Introduction

Valproic acid (VPA) is widely used as an antiepileptic drug and is also prescribed for the treatment of manic state in bipolar disorder and for prevention of migraine. VPA treatment during pregnancy, however, increases the risk of autism spectrum disorder (ASD) and attention-deficit/hyperactivity disorder (ADHD) in offspring^1,2^. A dose-dependent association with lower IQ in children exposed to VPA in utero has been demonstrated^3^. Although foetal VPA exposure has a detrimental effect on neurodevelopment, VPA continues to be in common use, because of the need to balance its detrimental effects with the risks of uncontrolled seizures or mood disorder. The mechanisms underlying the impact of VPA on neurodevelopment remain unclear. Understanding these mechanisms is essential to prevent VPA-induced detrimental events.

Prenatal VPA exposure induces social behaviour deficits, repetitive behaviours and cognitive impairment in mice^4,5^. Postnatal day 14 in rat roughly corresponds to the third trimester of human development^6^. Early postnatal VPA exposure in mice (on postnatal day 14) also induces social behaviour deficit and memory impairment^7,8^. Prenatal VPA exposure enhances glutamatergic synaptic transmission^9^ and impairs GABAergic synaptic transmission^10^, suggesting that the synaptic excitation/inhibition (E/I) ratio is increased, which may induce the behavioural changes. Direct VPA exposure of cultured cortical neurons isolated on embryonic day 15 causes an increased E/I ratio^11^. In addition, VPA exposure of cultured cortical neurons derived from postnatal day 1 rats suppresses the formation of GABAergic synapses, but not glutamatergic synapses^12^. It is clear that VPA exposure has an effect on neuronal development. However, it is unknown whether VPA exposure of astrocytes, which play important roles in synapse formation and in modulation of synaptic function, has an effect on neurodevelopment.

Astrocytes are the most abundant cell type in most parts of the brain^13^. During cortical development, neural stem cells first generate neurons, followed by astrocytes, and finally oligodendrocytes. Astrocyte formation occurs during late prenatal and early postnatal stages in rats^14^. Synapses are generated concurrently with astrocyte development during the early postnatal period^15^. Astrocytes regulate synaptogenesis and synaptic strength. In addition, effects of astrocytes on synapses are different between glutamatergic and GABAergic neurons^16–23^. Therefore, astrocytes are likely to play a key role in regulating the E/I ratio.

In this study, we investigated the effect of VPA-exposed astrocytes on synaptic properties using biochemical, cell biological and electrophysiological approaches. We found changes to the synaptic function of cortical inhibitory, but not excitatory, neurons co-cultured with VPA-exposed astrocytes.

## Results

### Inhibitory synaptic transmission is attenuated by VPA-exposed astrocytes

**Figure 1:**
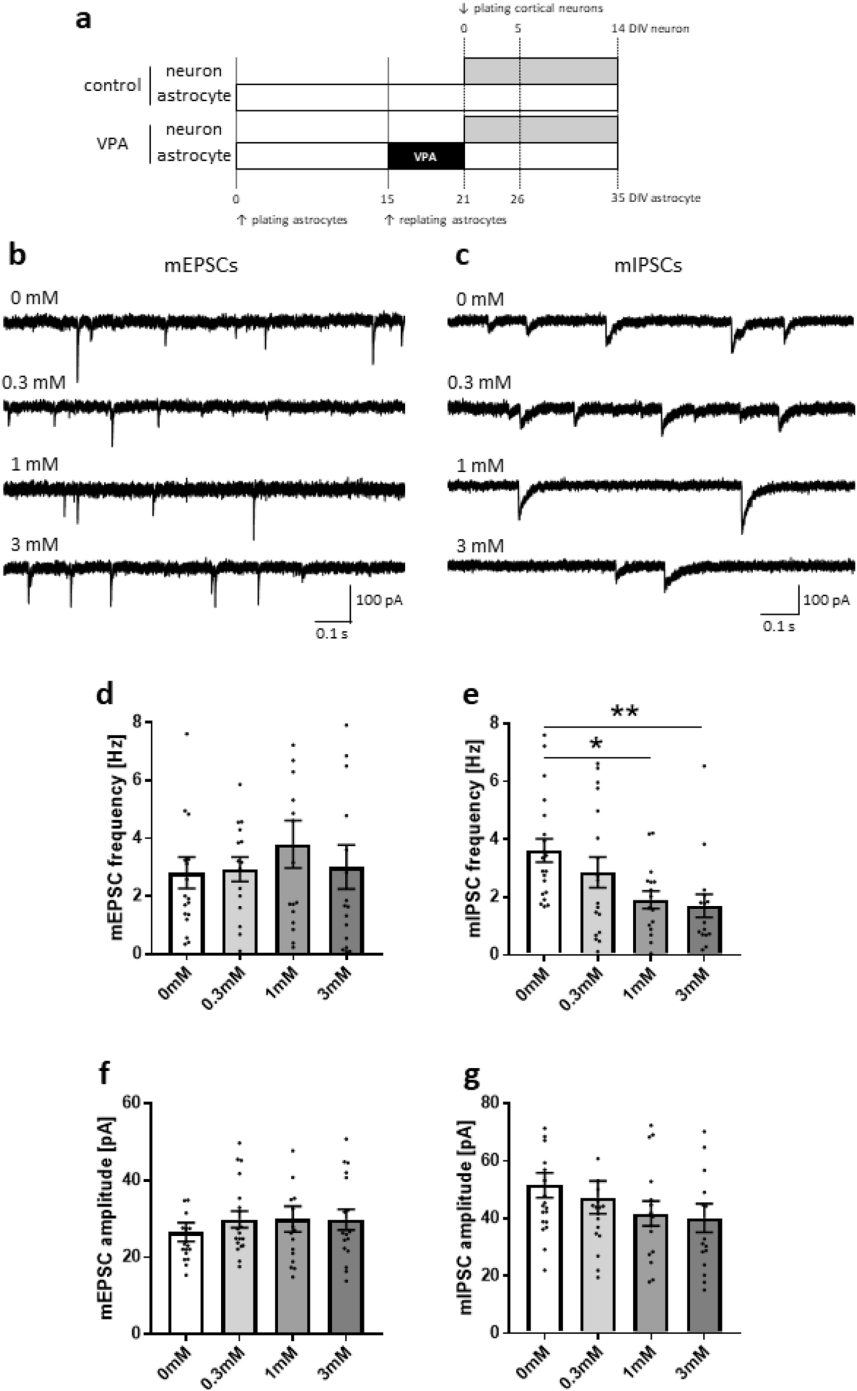
VPA-exposed astrocytes decrease inhibitory synaptic transmission. (**a**) Scheme for the procedure of plating cells and VPA exposure. (**b**,**c**) Representative traces of mEPSCs and mIPSCs were recorded in neurons co-cultured with control astrocytes and VPA-exposed astrocytes in the presence of 1 µM TTX and 2 µM gabazine or 5 µM CNQX plus 25 µM AP5, respectively. Before astrocytes were co-cultured with neurons, astrocytes were exposed to VPA at the following concentrations: 0.3 mM, 1 mM and 3 mM. (**d**) The frequency of mEPSCs in neurons co-cultured with VPA-exposed astrocytes was unchanged relative to neurons co-cultured with control astrocytes. (**e**) The frequency of mIPSCs in neurons co-cultured with VPA-exposed astrocytes was significantly decreased compared with neurons co-cultured with control astrocytes. (**f**,**g**) mEPSCs and mIPSCs amplitudes were not changed between neurons co-cultured with control astrocytes and VPA-exposed astrocytes. [n = number of neurons used to determine mEPSC frequency (**d**) and amplitude (**f**): 0 mM; n = 18, 0.3 mM; n = 15, 1 mM; n = 17, 3 mM; n = 17; mIPSC frequency (**e**) and amplitude (**g**): 0 mM; n = 20, 0.3 mM; n = 18, 1 mM; n = 16, 3 mM; n = 16; 8 cultures/4 experiments], *p < 0.05, **p < 0.01.

To assess the impact of VPA-exposed astrocytes on synaptic transmission of co-cultured neurons, we used whole-cell patch clamp recording under voltage clamp conditions. Of importance, neurons were not significantly exposed to VPA during the culture period but co-cultured astrocytes were (Fig. 1a). This method therefore enables the effect of VPA on astrocyte-mediated neuronal function to be evaluated. Patch clamps were performed on mass culture preparations and compared among four groups of astrocytes exposed to different concentrations of VPA (0 mM, 0.3 mM, 1 mM and 3 mM). We recorded miniature excitatory postsynaptic currents (mEPSCs) and miniature inhibitory postsynaptic currents (mIPSCs) as spontaneous synaptic network activity from cortical neurons at days in vitro (DIV) 14 (Fig. 1b, 1c). The frequency of mEPSCs in cultures with VPA-exposed astrocytes was comparable to that in control cultures (0 mM, 2.81 ± 0.54 Hz; 0.3 mM, 2.93 ± 0.42 Hz; 1 mM, 3.79 ± 0.81 Hz; 3 mM, 3.01 ± 0.76 Hz; Fig. 1d). However, the frequency of mIPSCs of neurons co-cultured with 1 mM VPA-exposed astrocytes was 53% of the control value. With 3 mM VPA-exposed astrocytes, the frequency of mIPSCs was 47% of the control value (0 mM, 3.61 ± 0.40 Hz; 0.3 mM, 2.86 ± 0.53 Hz; 1 mM, 1.91 ± 0.31 Hz; 3 mM, 1.70 ± 0.40 Hz; Fig. 1e). The amplitude of neither mEPSCs (0 mM, 26.6 ± 2.44 pA; 0.3 mM, 29.8 ± 2.18 pA, 1 mM, 29.9 ± 3.34 pA; 3 mM, 29.8 ± 2.69 pA; Fig. 1f) nor mIPSCs (0 mM, 51.6 ± 4.31 pA; 0.3 mM, 47.3 ± 5.72 pA, 1 mM, 41.7 ± 4.27 pA; 3 mM, 40.2 ± 5.03 pA; Fig. 1g) was affected by VPA-exposed astrocytes. These data indicated that VPA-exposed astrocytes attenuate inhibitory synaptic transmission of co-cultured neurons in a concentration-dependent manner. Given that VPA-exposed astrocytes attenuate inhibitory, but not excitatory, synaptic transmission, VPA may increase the E/I ratio via astrocytes.

### The number of GABAergic synapses is reduced by VPA-exposed astrocytes

**Figure 2:**
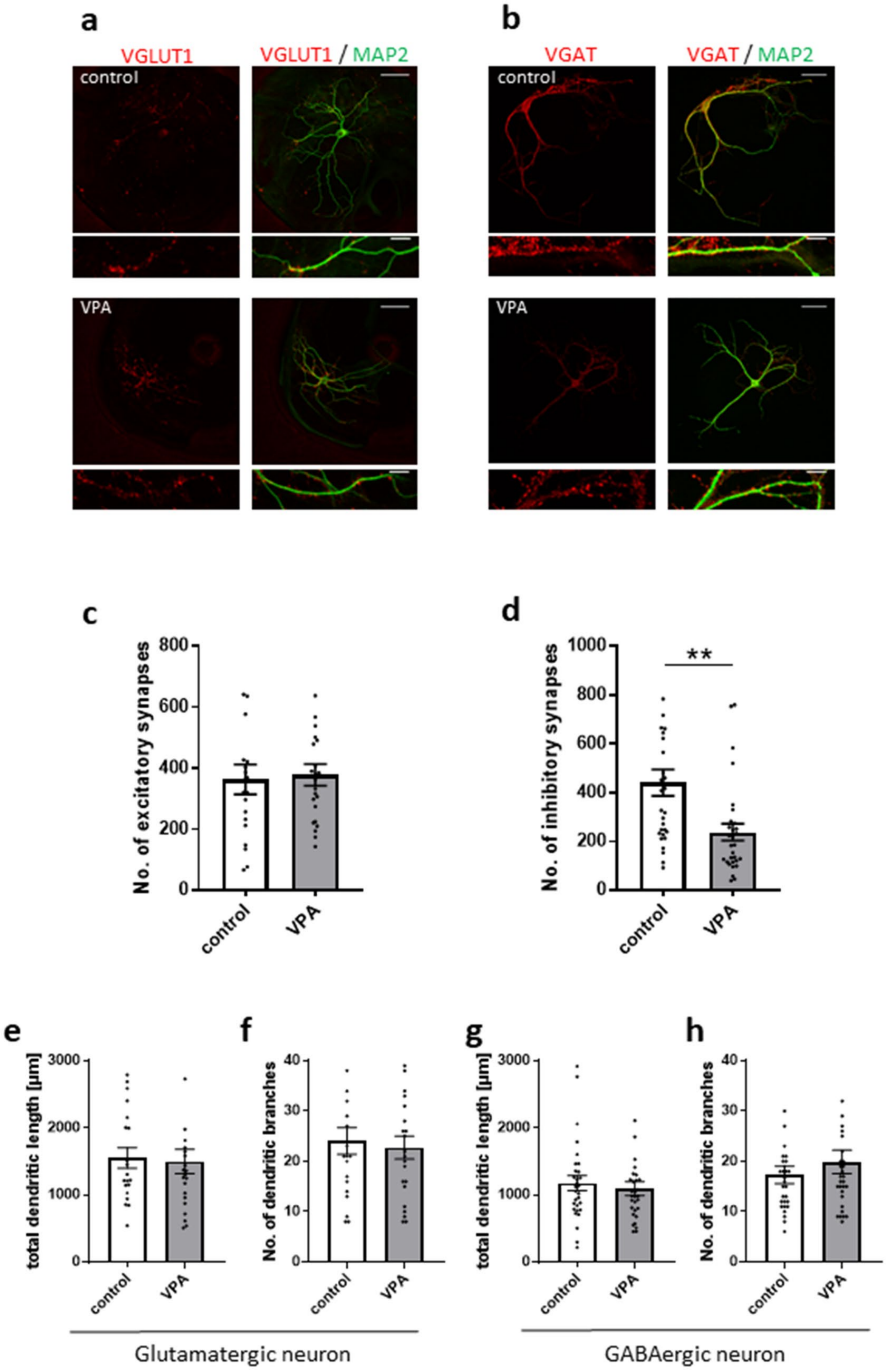
The number of VGAT-positive inhibitory synapses is decreased by VPA-exposed astrocytes without change in dendritic morphology. (**a**) Effects of VPA-exposed astrocytes on excitatory synapses and dendritic morphology. Representative images of autaptic neurons immunostained for the dendritic marker, microtubule-associated protein 2 (MAP2) (in green), and the excitatory synapse marker, vesicular glutamate transporter 1 (VGLUT1) (in red). (**b**) Effects of VPA-exposed astrocytes on inhibitory synapses and dendritic morphology. Representative images of autaptic neurons immunostained for the dendritic marker, MAP2 (in green), and the inhibitory synapse marker, vesicular GABA transporter (VGAT) (in red). Parts of the images in the top row (scale bars = 50 µm) are enlarged in the bottom row (scale bars = 10 µm). (**c**) The number of excitatory synapses (VGLUT1-positive) in neurons co-cultured with VPA-exposed astrocytes was unchanged relative to neurons co-cultured with control astrocytes (n = number of neurons: control, n = 20; VPA, n = 23). (**d**) The number of inhibitory synapses (VGAT-positive) in neurons co-cultured with VPA-exposed astrocytes was significantly decreased compared with neurons co-cultured with control astrocytes (n = number of neurons: control, n = 30; VPA, n = 30), **p < 0.01. (**e**,**f**) In glutamatergic neurons, total dendritic length and the number of branches were unchanged between neurons co-cultured with control astrocytes and VPA-exposed astrocytes (n = number of neurons: control, n = 20; VPA, n = 23). (**g**,**h**) In GABAergic neurons, total dendritic length and the number of branches were unchanged between neurons co-cultured with control astrocytes and VPA-exposed astrocytes (n = number of neurons: control, n = 30; VPA, n = 30). All data in Fig. 2 were obtained from 10 cultures/4 experiments.

The smaller mIPSC frequency without affecting amplitude in neurons co-cultured with VPA-exposed astrocytes indicated that VPA-exposed astrocytes may exhibit a small number of GABAergic synapses. To address this possibility, immunostaining for vesicular glutamate transporter 1 (VGLUT1) and vesicular GABA transporter (VGAT), which are glutamatergic and GABAergic presynaptic proteins, respectively, was performed (Fig. 2a,b). To count the number of presynaptic puncta in single neurons, an autaptic neuron culture system was used. Cortical neurons at DIV 14 were used. The number of VGAT-labelled puncta was significantly smaller in VGAT-positive neurons cultured with VPA-exposed astrocytes (control, 422.42 ± 46.23; VPA, 207.12 ± 30.35; Fig. 2d). However, the number of VGLUT1-labelled puncta was identical between VGLUT1-positive neurons cultured with control astrocytes and VPA-exposed astrocytes (control, 363.1 ± 48.97; VPA, 377.83 ± 35.26; Fig. 2c). It was consistent with the electrophysiological results that presynaptic terminals grew in glutamatergic but not GABAergic neurons. These results indicate that the smaller inhibitory synaptic transmission may be because of fewer GABAergic synapses.

### VPA-exposed astrocytes do not change dendritic morphology

Astrocytes with neurodevelopmental disorder-related mutations induce changes in dendritic morphology of co-cultured neurons^24,25^. Therefore, we analysed the length and branches of dendrites of glutamatergic and GABAergic cortical neurons at DIV 14 labelled by Microtubule-associated protein 2 (MAP2) (Fig. 2a,b). Neither dendritic length (control, 1550.56 ± 151.89 µm; VPA, 1496.84 ± 179.92 µm; Fig. 2e) nor the number of branches (control, 24.05 ± 2.64; VPA, 22.73 ± 2.30; Fig. 2f) of VGLUT1-positive neurons were affected by VPA-exposed astrocytes. Likewise, dendrite length (control, 1175.65 ± 110.42 µm; VPA, 1094.68 ± 103.66 µm; Fig. 2g) was similar between VGAT-positive neurons cultured with control astrocytes and VPA-exposed astrocytes. In addition, there was no difference in the number of dendritic branches (control, 17.3 ± 1.72; VPA, 19.87 ± 2.32; Fig. 2h). These data indicate that the VPA-exposed astrocytes did not affect dendritic morphology of glutamatergic and GABAergic co-cultured neurons.

### VPA-exposed astrocytes suppress GABAergic synapse development without affecting axon growth

**Figure 3:**
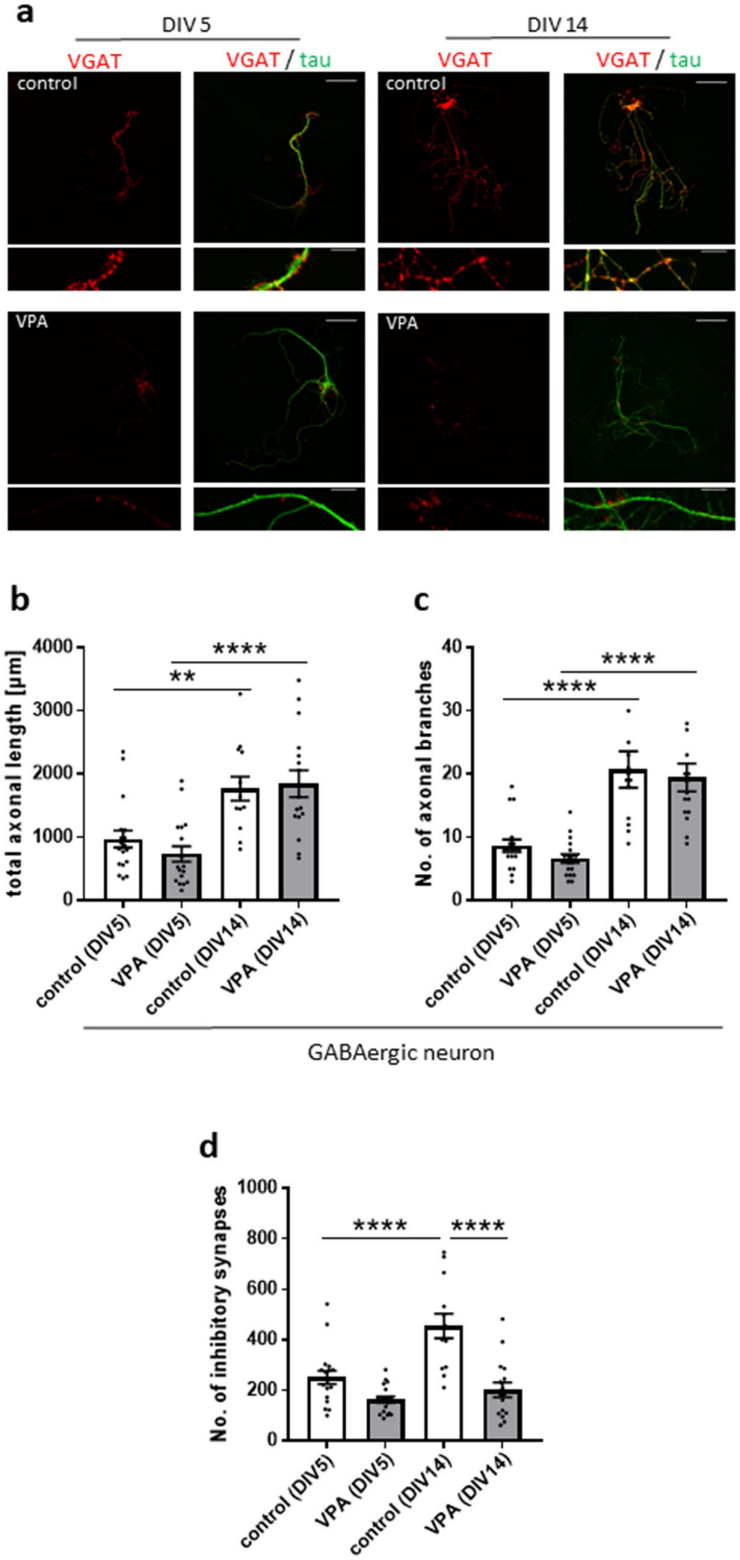
VPA-exposed astrocytes suppress GABAergic synapse development without affecting axonal growth. (**a**) Effects of VPA-exposed astrocytes on inhibitory synapses and axonal morphology. Representative images of autaptic neurons immunostained for the axonal marker, tau (in green), and vesicular GABA transporter, (VGAT) (in red). Axon morphology and synapse formation were analysed at early (DIV 5) and late (DIV 14) phases. Parts of the images in the top row (scale bars = 50 µm) are enlarged in the bottom row (scale bars = 10 µm). (**b**,**c**) Total axon length and the number of axon branches were increased from DIV 5 to DIV 14 in both neurons with control and VPA-exposed astrocytes. There were no differences in axon length and the number of axon branches between neurons with control astrocytes and VPA-exposed astrocytes. (**d**) The number of GABAergic synapses (VGAT-positive) in neurons co-cultured with control astrocytes was significantly increased from DIV 5 to DIV 14, but not in neurons co-cultured with VPA-exposed astrocytes. [**b**–**d**: n = number of neurons: control (DIV 5), n = 18; VPA (DIV 5), n = 18; 2 cultures/1 experiment; control (DIV 14), n = 13; VPA (DIV 14), n = 16; 2 cultures/1 experiment], **p < 0.01, ****p < 0.0001.

Astrocyte-released proteins selectively enhance GABAergic axon length, branching and synapse formation^17^. To examine whether attenuation of GABAergic synapse formation is caused by reduced axon growth, we examined axon morphology and synapse formation at early (DIV 5) and late (DIV 14) phases. We analysed axon length and branches labelled by a tau antibody (Fig. 3a). As a result, axon length and the number of axon branches increased from the early to the late phase in both GABAergic neurons with control astrocytes and VPA-exposed astrocytes. In turn, there were no differences in axon length or in the number of axon branches between GABAergic neurons with control astrocytes and VPA-exposed astrocytes [length: control (DIV 5), 973.62 ± 135.81 µm; VPA (DIV 5), 737.87 ± 121.58 µm; control (DIV 14), 1764.71 ± 191.85 µm; VPA (DIV14), 1845.51 ± 213.162 µm; Fig. 3b; the number of branches: control (DIV 5), 8.67 ± 0.97; VPA (DIV 5), 6.67 ± 0.69; control (DIV 14), 20.69 ± 2.88; VPA (DIV 14), 19.44 ± 2.22; Fig. 3c]. VGAT-positive neurons with control astrocytes showed a developmental increase in the number of VGAT-labelled puncta, while neurons with VPA-exposed astrocytes did not show such an increase in the number of VGAT-labelled puncta [control (DIV 5), 251.11 ± 26.25; VPA (DIV 5), 163.39 ± 13.22; control (DIV 14), 455.00 ± 48.85; VPA (DIV 14), 202.19 ± 29.33; Fig. 3d]. Although GABAergic synapse development was suppressed in neurons with VPA-exposed astrocytes, axon growth in neurons with VPA-exposed astrocytes was identical to that in neurons with control astrocytes. These results indicated that VPA-exposed astrocytes attenuate GABAergic synapse formation, independent of axon growth.

### VPA-exposed astrocytes reduce *Ptprd* mRNA levels in co-cultured neurons

Synapse formation occurs through signalling mediated by cell surface molecules at developing presynaptic terminals and postsynaptic membranes. As examples, Semaphorin4D is selectively required for proper GABAergic synapse development but not glutamatergic synapse development^26,27^. Semaphorin4D-Fc treatment increases GABAergic synapse formation in a Plexin-B1-dependent manner^28^. Trans-synaptic interaction between Slit and NTRK-like family member 3 (SLITRK3) and protein tyrosine phosphatase receptor type delta (PTPRD) induces only GABAergic presynaptic differentiation^29,30^. SLITRK3-deficient mice show decreased mIPSC frequency with no change in mIPSC amplitude^29^. The Contactin5/Caspr4 complex plays a role in GABAergic synaptogenesis in the spinal cord^31^. According to these critical findings, it was speculated that VPA-exposed astrocytes attenuated GABAergic synapse formation. We, therefore, analysed the mRNA levels of *Semaphorin4D (Sema4D)*, *Plexin-B1 (PlxnB1)*, *Slitrk3*, *Ptprd*, *Contactin5 (Cntn5)* and *Caspr4*, which are required for the development of GABAergic synapses.

**Figure 4:**
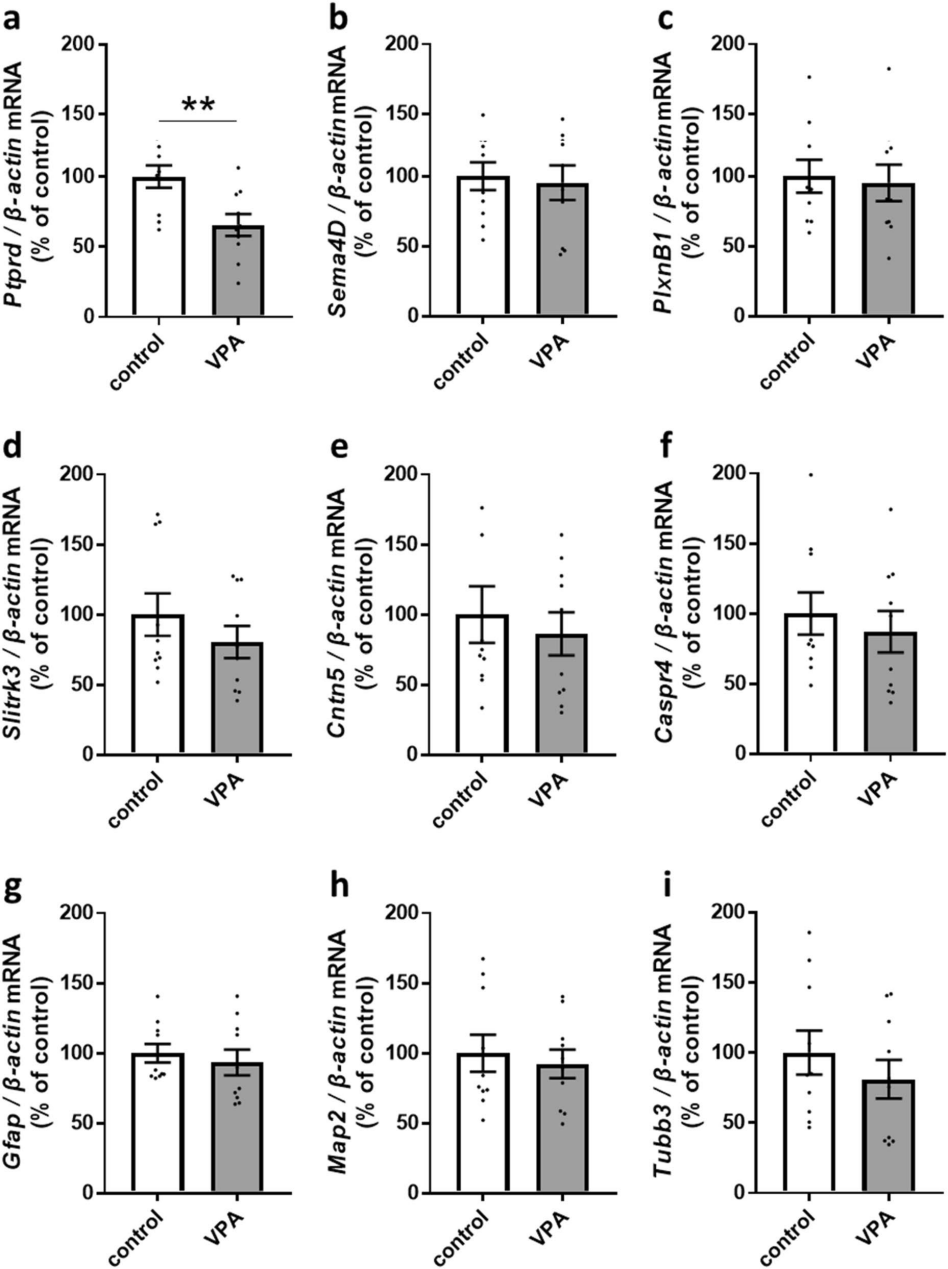
VPA-exposed astrocytes exhibited reduced *Ptprd* mRNA levels in co-cultured neurons at DIV 14. (**a–f**) mRNA levels of cell surface molecules in GABAergic synapses and (**g**–**i**) mRNA levels of *Gfap* and *Map2*, and *Tubb3* in neurons co-cultured with control astrocytes or VPA-exposed astrocytes (n = number of neurons: control, n = 10; VPA, n = 10; 10 cultures/2 experiments). mRNA from cortical neurons with astrocytes was harvested at DIV 14.

Neurons co-cultured with VPA-exposed astrocytes showed significantly reduced levels of *Ptprd* mRNA at DIV 14 (control, 100 ± 7.92%; VPA, 65.5 ± 7.84%, Fig. 4a), but the mRNA levels of other molecules were unchanged (*Sema4D*: control, 100 ± 9.98%, VPA, 95.26 ± 12.33%, Fig. 4b; *PlxnB1*: control, 100 ± 11.74%, VPA, 95.27 ± 13.08%, Fig. 4c; *Slitrk3*: control, 100 ± 15.10%, VPA, 80.54 ± 11.35%, Fig. 4d; *Cntn5*: control, 100 ± 20.12%, VPA, 86.31 ± 15.29%, Fig. 4e; *Caspr4*: control, 100 ± 14.98%, VPA, 87.01 ± 14.8%, Fig. 4f). At DIV 7, the mRNA levels of all molecules including *Ptprd* were unchanged (Supplementary Fig. S2a–f). Given that the number of VGAT-labelled puncta in neurons co-cultured with VPA-exposed astrocytes was decreased at DIV 14 but not DIV 5, these data indicated that VPA-exposed astrocytes may cause dysplasia of GABAergic synapses by suppressing the level of *Ptprd* mRNA during the development of co-cultured inhibitory neurons. *Gfap* mRNA levels are not different between neurons co-cultured with control astrocytes or VPA-exposed astrocytes, indicating that VPA exposure had no effect on proliferation or differentiation of astrocytes (DIV 7: Supplementary Fig. S2g; DIV 14: control, 100 ± 6.68%, VPA, 93.3 ± 9.19%; Fig. 4g). Similarly, there was no change in *Map2* or *Tubb3* mRNA levels, demonstrating that VPA-exposed astrocytes did not affect dendrite or axon growth (DIV 7: Supplementary Fig. S2h, i; DIV 14: *Map2*: control, 100 ± 13.16%, VPA, 92.3 ± 10.1%, Fig. 4h; *Tubb3*: control, 100 ± 15.71%, VPA, 81.09 ± 13.8%; Fig. 4i).

## Discussion

We investigated whether VPA-exposed astrocytes affect synaptic properties of co-cultured neurons. We demonstrated that VPA-exposed astrocytes impair GABAergic, but not glutamatergic, synapse formation and synaptic transmission. These results indicate that VPA-exposed astrocytes induce E/I imbalance in local neuronal networks.

An important conclusion from this study is that VPA indirectly changes GABAergic presynaptic properties via astrocytes. GABAergic pre- and postsynaptic functions are impaired in the offspring of VPA-treated female rats^10^. VPA-exposed astrocytes possibly cause the presynaptic impairment in the offspring. Cultured neurons directly exposed to VPA also demonstrate reduced GABAergic synapse formation and function^11,12^. Therefore, VPA may influence both neurons and astrocytes to cause impairment of GABAergic function by additive or synergistic effects.

VPA-exposed astrocytes exhibited smaller GABAergic synaptic transmission while glutamatergic synaptic transmission was unchanged. This result indicates that VPA causes E/I imbalance in neuronal networks via astrocyte-neuron interaction. E/I imbalance is observed in animal models of autism and schizophrenia^32–34^. Therefore, E/I imbalance is thought to be part of the neuropathology of autism and schizophrenia. Given that VPA-exposed astrocytes cause E/I imbalance, neurological and neurodevelopmental disorders, such as autism and schizophrenia, may result from abnormal synapse formation induced by astrocytes.

GABAergic neurons co-cultured with VPA-exposed astrocytes showed low frequency of mIPSCs without a change in amplitude, indicating that VPA-exposed astrocytes impair presynaptic function in GABAergic neurons. To analyse change in presynaptic terminals, we examined expression of VGAT by immunocytochemistry. The number of VGAT-positive puncta in neurons co-cultured with VPA-exposed astrocytes was smaller than that in neurons co-cultured with control astrocytes. These results indicate that deficiency of VGAT-positive presynapses causes presynaptic dysfunction.

PTPRD is expressed in excitatory and inhibitory presynapses. Presynaptic PTPRD forms trans-synaptic adhesion complexes with multiple postsynaptic binding partners, including Interleukin 1 receptor accessory protein-like 1 (IL1RAPL1)^35,36^, Interleukin 1 receptor accessory protein (IL-1RAcP)^37^ and Slit- and Trk-like proteins (SLITRKS)^30^, to regulate synapse formation. PTPRD interacts with SLITRK3 in GABAergic synapses, and the GABAergic-specific trans-synaptic adhesion complex is required for GABAergic synapse formation^29^. In this study, *Ptprd* mRNA levels were decreased but *Slitrk3* mRNA levels were not changed. PTPRD knockdown in cultured hippocampal neurons causes a reduction in GABAergic synapse formation and transmission without affecting glutamatergic synapse formation and transmission^38^, despite PTPRD being expressed in both synapses. Therefore, the altered GABAergic synapse formation and synaptic transmission in this study may be caused by a reduced level of PTPRD.

The neurodevelopmental disorder Rett syndrome, which is an X-linked ASD, is caused by mutation in the transcription factor, methyl-CpG-binding protein 2 (MeCP2). MeCP2-deficient astrocytes fail to support normal dendritic morphology in healthy hippocampal neurons^24^. Fragile X syndrome, which is a leading form of ASD, is caused by mutation in the promoter region of the fragile X mental retardation 1 gene (*FMR1*). *Fmr1* KO astrocytes alter the dendritic morphology of healthy cortical neurons^25^. These reports demonstrate that ASD model-derived astrocytes contribute to abnormal neuronal dendrite development. In this study, VPA-exposed astrocytes did not alter dendrite morphology of glutamatergic or GABAergic neurons. However, defects in GABAergic presynapses are caused by co-culture with VPA-exposed astrocytes. VPA-exposed astrocytes may therefore be implicated in neurodevelopmental disorders through mechanisms that are different to those in Rett and Fragile X syndromes. VPA increases mRNA levels of cell adhesion (neuroligin 1 and neuregulin 1) and extracellular matrices molecules (neuronal pentraxin 1 and thrombospondin 3) in primary astrocyte cultures^39^. Rats prenatally exposed to VPA show changes in expression of GLT1, which is highly expressed in astrocytes and takes up glutamate at the synaptic cleft^40^. However, in astrocytes, none of the molecules mentioned above were known to regulate GABAergic synapse formation until now. Astrocytes modulate synapse formation and function through contact-mediated signalling and secreted signalling factors. Transforming growth factor β derived from astrocytes induces synaptogenesis of excitatory and inhibitory neurons^16,19^. However, thrombospondins do not substantially contribute to GABAergic synaptogenesis, although thrombospondins are necessary and sufficient to induce glutamatergic synaptogenesis^17^. Cholesterol also enhances presynaptic formation of retinal ganglion cells^41^ but not GABAergic synapse formation^42^. Brain-derived neurotrophic factor (BDNF) is produced by neurons and astrocytes^43^. Astrocyte-secreted BDNF does not affect GABAergic synaptogenesis in neuron-astrocyte co-cultures, while neuron-secreted BDNF and TrkB signalling between neurons promote formation of GABAergic synapses^42,44^. Neuronal neuroligin 2, which is an adhesion molecule, is expressed in GABAergic postsynaptic membranes and mediates GABAergic synapse formation and function^45,46^. Astrocytes also express neuroligin 2. A lack of neuroligin 2 in astrocytes diminishes the formation and function of excitatory synapses and enhances the function of inhibitory synapses; however, a lack of neuroligin 2 in astrocytes did not affect the formation of inhibitory synapses^18^. As mentioned above, although astrocytic factors are reported to modulate synapse formation and function in excitatory and inhibitory neurons, the existence of astrocytic factors that specifically modulate synaptogenesis in inhibitory neurons remains unknown. We aim to identify molecules in VPA-exposed astrocytes that affect GABAergic synapse properties in future studies.

To examine astrocytes in VPA-induced neurodevelopmental failure, we investigated the effect of VPA-exposed astrocytes on synapse development in a neuron-astrocyte co-culture system. We demonstrated that VPA indirectly suppresses GABAergic synapse formation via astrocytes. qRT-PCR experiments showed reduced levels of *Ptprd* mRNA in neurons. Suppression of PTPRD expression reduces GABAergic synapse formation and transmission [30.38]. In addition, mutations in PTPRD have been identified in individuals with ASD and ADHD^47,48^. VPA treatment during pregnancy increases the risk of ASD and ADHD in offspring^1,2^. Therefore, PTPRD may play a key role in the neuropathology of ASD and ADHD induced by prenatal VPA exposure. Taken together, our study suggests that VPA-exposed astrocytes may contribute to the development of VPA-induced neurodevelopmental failure.

## Methods

### Animals

All procedures for animal experimentation were carried out in strict accordance with the rules of the Experimental Animal Care and Welfare Committee of Fukuoka University, following approval of the experimental protocol (Permit Numbers: 1806022). ICR mice on day 12 of pregnancy were purchased from CLEA Japan (Tokyo, Japan). Pregnant mice were individually housed in plastic cages at a temperature of 23 ± 2 °C with a relative humidity of 60 ± 10% under a 12 h light–dark cycle (lights on 07:00–19:00). Food and water were available ad libitum. Newborn (postnatal day 0–1) mice were used for primary cultures.

### Primary astrocyte cultures

Mass and microisland preparations of cortical astrocytes were cultured as previously reported^49–51^. Cerebral cortices were isolated from the brain of newborn mice, and cells were then plated in culture flasks. After 15 days, when the culture was confluent, the microglia and other small cells were removed by tapping the culture flask. Cells adhering to the culture flasks were replated on either coverslips prepared for mass culture, at a density of 24,000 cells/cm^2^, or on dot-stamped coverslips prepared for microisland culture, at a density of 6,000 cells/cm^2^.

### Co-culture of cortical neurons and astrocytes

Mass and microisland preparations of cortical neurons were cultured as previously reported^49–51^. Cortical neurons were plated on astrocyte microislands and layers 6 days after the astrocytes were replated. Cerebral cortices were isolated from the brains of newborn mice and enzymically dissociated for 55 min at 37 °C. The conditioned medium of the mass and microisland astrocyte cultures was replaced with medium for neuron cultures, and neurons were plated. For autaptic neuron culture, cells were plated at a density of 3,000 cells/cm^2^ onto the astrocyte microislands. For mass neuron culture, cells were plated at a density of 13,000 cells/cm^2^ onto the astrocyte layer. Cortical neurons were co-cultured for 13–14 days on microisland astrocytes or mass cultured astrocytes.

### VPA treatment

VPA (1 M, Fujifilm Wako Pure Chemical Corporation, Osaka, Japan) was prepared in sterile water. VPA solution was diluted from 1 M to 0.3, 1, and 3 mM with astrocyte medium. Astrocytes were exposed to VPA (0.3, 1, and 3 mM) for 6 days after replating on coverslips. Then, medium was removed and cells were maintained in normal neuron growth medium. We examined the residual VPA levels in normal neuron growth medium using liquid chromatography-tandem mass spectrometry (Supplementary Fig. S1). The residual levels (0.011 ± 0.0007 mM, n = 3) were lower than the level of VPA (0.3 mM) that was previously reported to not affect development of GABAergic neurons^12^.

### Immunocytochemistry, image acquisition and analysis

Immunostaining was performed as previously reported^50,51^. After fixation, permeabilization and blocking, autaptic neurons cultures were incubated with the following primary antibodies: anti-MAP2 (guineapig polyclonal, Synaptic Systems, Goettingen, Germany, Cat. No. 188 004, 1:1000 dilution)^50^, anti-VGLUT1 (rabbit polyclonal, Synaptic Systems, Cat. No. 135 302, 1:2000 dilution)^50^, anti-VGAT (rabbit polyclonal, Synaptic Systems, Cat. No. 131 002, 1:1000 dilution), and anti-tau (mouse monoclonal, Cell Signaling Technology, Beverly, MA, USA, Cat. No. 4019, 1:1000 dilution). Then, samples were incubated with the appropriate species-specific fluorochrome-labelled goat secondary antibodies [Alexa Fluor 488 (Invitrogen, Carlsbad, CA, USA, Cat. No. A11073) for MAP2, Alexa Fluor 594 (Invitrogen, Cat. No. A11037) for VGLUT1 and VGAT, and CF 488A (Sigma-Aldrich, St. Louis, MO, USA, Cat. No. SAB4600042) for tau, 1:400 dilution]. Samples were mounted in mounting medium containing DAPI (ProLong Gold Antifade Mountant with DAPI, Invitrogen, Cat. No. P36931).

Confocal images of neurons were captured as previously reported^51^. Synaptic puncta were analysed according to previously reported procedures^50–52^. Dendritic and axonal morphologies were analysed using NeuronJ, which was accessed as an ImageJ plugin (v1.4.3, Erik Meijering).

### Electrophysiology

Mass neuron cultures (DIV 13 or 14) were used for whole-cell voltage-clamp electrophysiological recordings. The recordings were performed according to the previous reports^50,51^. Patch pipettes (2.5–5.0 MΩ) were filled with intracellular solution (mM): KCl 136.2, MgCl_2_ 0.6, ATP-Na_2_ 4, GTP-Na_2_ 0.3, phosphocreatine 12, EGTA 1, HEPES 17.8 and creatine phosphokinase 50 U/ml, pH 7.4. mEPSCs and mIPSCs were recorded at a holding potential (Vh) of –70 mV in the presence of 1 µM TTX and 2 µM gabazine (for mEPSCs) or 5 µM CNQX plus 25 µM AP5 (for mIPSCs).

### Quantitative RT-PCR

Quantitative RT-PCR analysis was performed according to the procedures described by Kanaoka et al.^53^. At DIV 7 and 14, total RNA was extracted from co-cultured cells using the ISOSPIN Cell & Tissue RNA (Nippon Gene, Tokyo, Japan). Quantitative PCR was conducted on a LightCycler Nano System (Roche Diagnostics, Mannheim, Germany) using primers (see Table 1). Analysis of PCR data was performed using the comparative Ct method.

**Table 1:** Oligonucleotide sequences of quantitative RT-PCR primer sets.

Primer	Sequence (5′-3′)
(Forward)	
(Reverse)	
PTPRD-F	5′-CCC CCA GGT TTA CAC GAA CTC-3′
PTPRD-R	5′-ATC CAG ACC CAT CGT CAA ATT C-3′
Sema4D-F	5′-CCT TGA GGA CGG AGT ATG CC-3′
Sema4D-R	5′-TCT GGA TCA CGT CAG CAA AGA-3′
PlxnB1-F	5′-CAC ACA TCT ACT ACA CTT GGC AA-3′
PlxnB1-R	5′-CAA TCC CGG CTG TCA TTC AC-3′
Slitrk3-F	5′-TGA AGC CAA GCA TAG CTG AAA-3′
Slitrk3-R	5′-ATC AGG GGA ATT GGG GTA GTC-3′
Cntn5-F	5′-ACT CCT CAG ATG CCT TCA GAC A-3′
Cntn5-R	5′-AGT TCC ATT CCG AAG CCA TCT G-3′
Caspr4-F	5′-TTT GGA ACG CAG CTT CCT TTA-3′
Caspr4-R	5′-GAG AGG GCT GTC GTC TTG AAA-3′
GFAP-F	5′-GCA AAA GCA CCA AAG AAG GGG A-3′
GFAP-R	5′-ACA TGG TTC AGT CCC TTA GAG G-3′
MAP2-F	5′-GCC AGC CTC AGA ACA AAC AG-3′
MAP2-R	5′-AAG GTC TTG GGA GGG AAG AAC-3′
Tubb3-F	5′-TAG ACC CCA GCG GCA ACT AT-3′
Tubb3-R	5′-GTT CCA GGT TCC AAG TCC ACC-3′
β-actin-F	5′-GGC TGT ATT CCC CTC CAT CG-3′
β-actin-R	5′-CCA GTT GGT AAC AAT GCC ATG T-3′

### Statistical analysis

All statistical tests were performed using GraphPad Prism 7 software (San Diego, CA, USA). To compare two groups (control vs. VPA), we used an unpaired Student’s t-test, and for multiple comparisons, we used a one-way ANOVA followed by Tukey’s (Fig. 3) or Dunnett’s test (Fig. 1). The results are expressed as the mean ± standard error of the mean (SEM), and p values less than 0.05 were considered significant.

### Data availability

The datasets generated during and/or analysed during the current study are available from the corresponding author on reasonable request.

## Supplementary Information

Supplementary Information 1.
