# Supplementary material for: Valproic acid-exposed astrocytes impair inhibitory synapse formation and function
Source: Sci Rep. 2021 Jan 8;11:23. doi: 10.1038/s41598-020-79520-7 (PMC7794250; doi:10.1038/s41598-020-79520-7)
Supplement: Supplementary file 1 — Supplementary Information 1. [file 41598_2020_79520_MOESM1_ESM.docx]

**Supplemental Information**

**Valproic acid-exposed astrocytes impair inhibitory synapse formation and function**

Kotomi Takeda, Takuya Watanabe, Kohei Oyabu, Shuntaro Tsukamoto, Yuki Oba, Takafumi Nakano, Kaori Kubota, Shutaro Katsurabayashi, Katsunori Iwasaki

**Supplemental methods**

**Liquid chromatography-tandem mass spectrometry**

Neuron growth medium (1000 µL) used for liquid chromatography-tandem mass spectrometry (LC-MS/MS) analysis was retrieved immediately after astrocyte medium was exchanged for neuron growth medium. After centrifugation (10,000 rpm, 10 min), 500 µL supernatant was transferred to a screw-top vial (Waters Corporation, Milford, MA, USA).

A standard stock solution of valproic acid (VPA; 1 mg/mL) was prepared by dissolving VPA in ultrapure water. The VPA working solutions for calibration and controls were prepared from the stock solution by adequately diluting the solution in medium. Consequently, five concentrations of VPA working solution were prepared (0.01, 0.1, 0.5, 1, and 10 µg/mL) for calibration.

The analytical platform comprised an LC system (ACQUITY Ultra Performance LC, Waters Corporation) coupled to an MS/MS spectrometer (Quattro Premier XE, Waters Corporation) equipped with Mass Lynx software version 4.1 (Waters Corporation). An ACQUITY UPLC BEH, C18 (2.1✕50 mm, 1.7 µm) analytical column (Waters Corporation) was used. The LC solvent program was operated at 40°C. The mobile phase comprised solvent A (5 mM ammonium acetate with 0.1% formic acid in water) and solvent B (acetonitrile/water (95/5, v/v)) at a constant flow rate of 0.4 mL/min. The LC eluate was introduced into a triple quadrupole MS/MS system equipped with an electro spray ionization source. The MS/MS system was operated in negative mode for VPA. The mass spectrometer was operated in negative ion multiple reaction monitoring (MRM) modes. The detection window was from 0.5 to 4 min of the chromatographic run. The collision cell gas comprised ultra-high purity argon supplied by Fukuoka Sanso Co. (Fukuoka, Japan).

**Supplemental Figures**


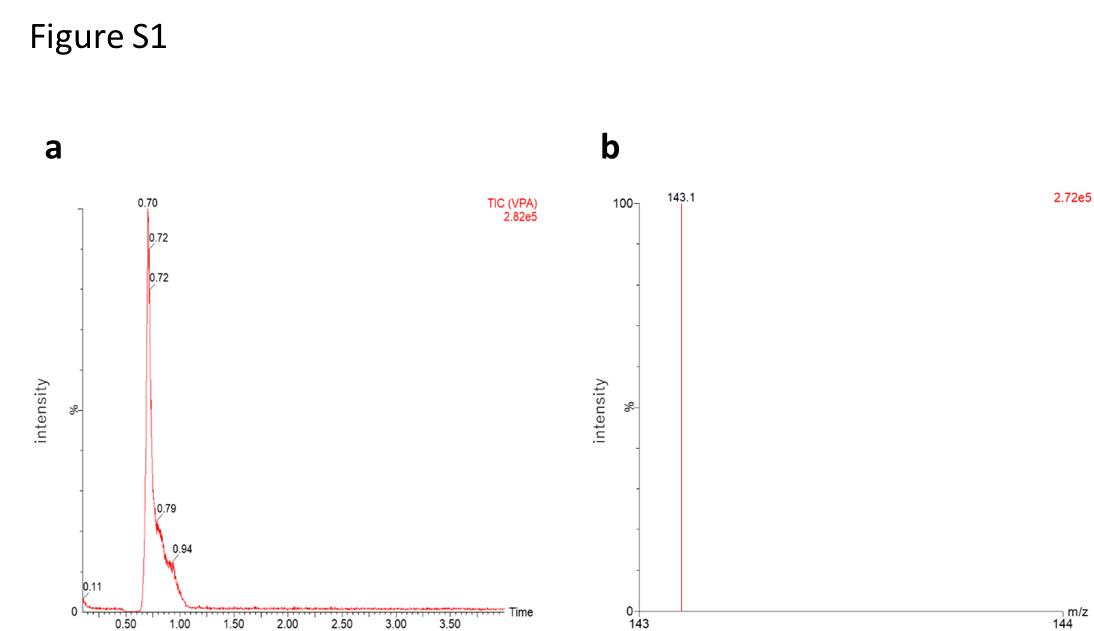


**Figure S1.**

Representative multiple reaction monitoring chromatogram (**a**) and mass spectra (**b**) of valproic acid (VPA). The concentration of VPA remaining in normal neuron growth medium was 0.011±0.0007 mM (n=3, 3 cultures/1 experiment).


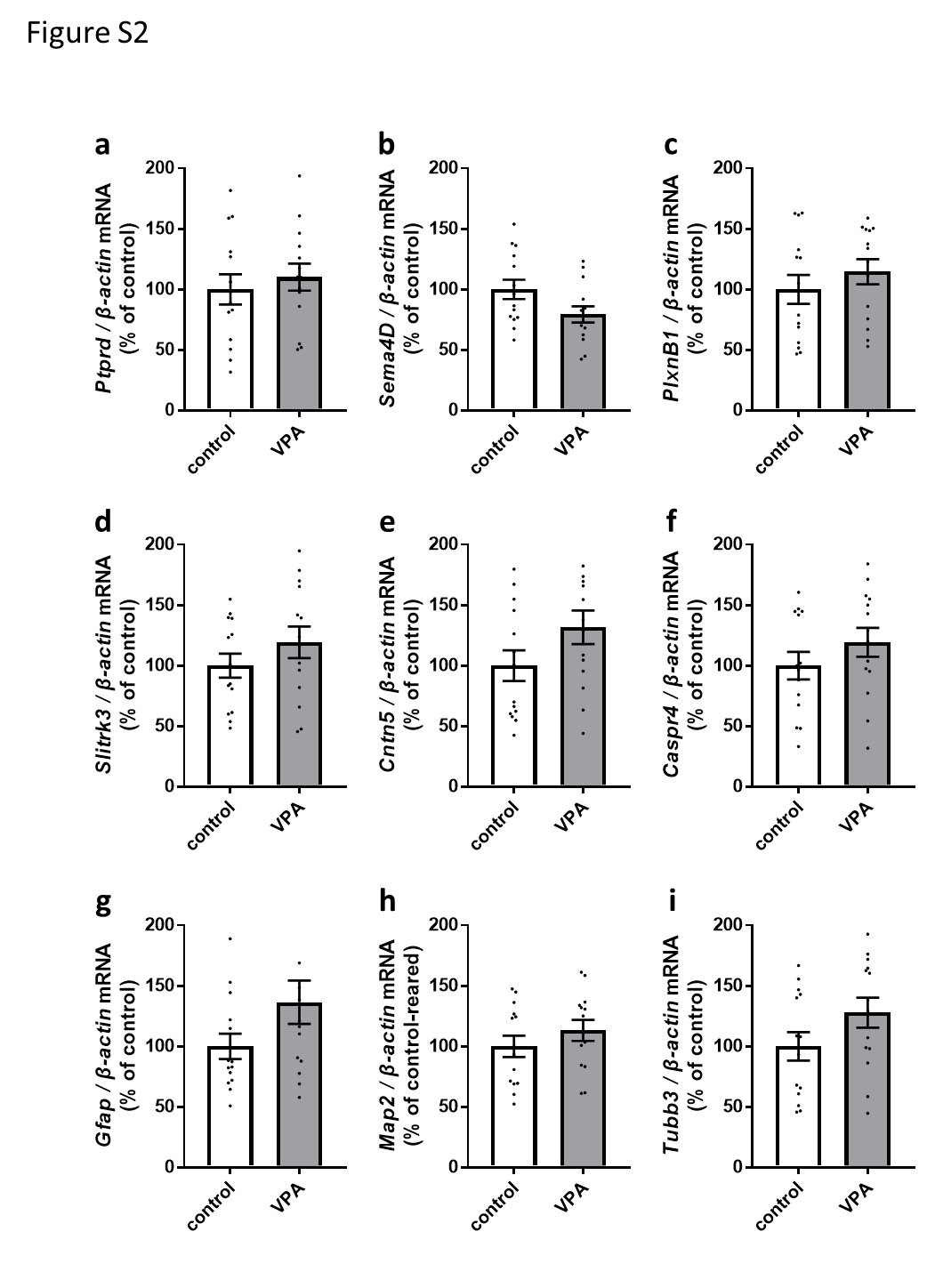


**Figure S2.**

Valproic acid (VPA)-exposed astrocytes exhibited unchanged *Ptprd* mRNA levels in co-cultured neurons at days *in vitro* (DIV) 7. (**a**) *Ptprd*: control, 100±12.53%; VPA, 110.2±11.15% (**b**) *Sema4D*: control, 100±8.03%; VPA, 79.32±6.672% (**c**) *Plxnb1*: control, 100±12.01%; VPA, 114.6±10.38% (**d**) *Slitrk3*: control, 100±9.926%; VPA, 119.3±12.95% (**e**) *Cntn5*: control, 100±12.62%; VPA, 131.7±13.87% (**f**) *Caspr4*: control, 100±11.3%; VPA, 119.4±12% (**g**) *Gfap*: control, 100±10.52%; VPA, 136.5±17.99% (**h**) *Map2*: control, 100±8.897%; VPA, 113.1±8.605% (**i**) *Tubb3*: control, 100±11.79%; VPA, 127.9±12.37%. mRNA from cortical neurons co-cultured with astrocytes was harvested at DIV 7 (control, n=14; VPA, n=14; 14 cultures/5 experiments).
